# How surface texture affects consumers’ willingness to pay: Evidence from smartphone covers

**DOI:** 10.1371/journal.pone.0338004

**Published:** 2025-12-04

**Authors:** Yoshihiko Kadoya, Mostafa Saidur Rahim Khan, Honoka Nabeshima, Yu Kuramoto, Yuichi Kurita

**Affiliations:** 1 School of Economics, Hiroshima University, Kagamiyama, Higashihiroshima, Japan; 2 School of Engineering, Hiroshima University, Kagamiyama, Higashihiroshima, Japan; Shandong University of Science and Technology, CHINA

## Abstract

This study investigates the impact of tactile impressions on consumers’ willingness to pay (WTP) for smartphone covers with varying surface textures. While prior research has examined visual and auditory cues in consumer behavior, the commercial relevance of surface texture remains underexplored. Therefore, we analyze how WTP varies across texture types and reference price conditions and examine the demographic, socioeconomic, and behavioral factors influencing valuation. Data were collected from 387 respondents at a major shopping mall, where participants evaluated four surface textures (A–D) under two reference prices (100 yen and 1,000 yen). Ordinary Least Squares (OLS) regression was used to assess associations between consumer characteristics and WTP. The results show that WTP increases with higher reference prices, indicating a price anchoring effect. Men, unmarried individuals, and those exhibiting hyperbolic discounting demonstrated higher WTP for certain textures. However, risk preference, smartphone usage, and impatience were not significant predictors. These findings suggest that firms can position textured products at premium price points and employ sensory marketing strategies, such as in-store trials, to enhance consumer engagement. This study contributes to the literature by integrating tactile marketing with behavioral economic theory, offering novel insights into joint influence of sensory cues and time preferences on consumer valuation.

## Introduction

Surface texture plays a critical role in product design by influencing consumer perceptions, experiences, and purchasing decisions. While visual and auditory cues are frequently considered in marketing and product development, tactile impressions remain an underexplored yet potentially significant factor in value creation [1–7]. Existing research in sensory marketing has demonstrated that product texture can affect user satisfaction, brand perception, perceived product quality, and emotional responses, all of which influence consumer preference [3,8–13]. However, the commercial viability of tactile impressions, particularly regarding consumers’ willingness to pay (WTP) for differentiated surface textures, has not been extensively explored. This study addresses this gap by examining how tactile impressions influence consumer behavior, extending prior research based on smaller, more homogeneous samples [14].

Previous studies suggest that consumers evaluate products through multisensory experiences and that tactile perception plays a crucial role in shaping preferences [9,10,15–18]. Product texture can trigger emotional responses, improve usability, and create associations between durability and quality [9,17,18]. Tactile impressions influence consumer behavior by increasing purchase intention and product satisfaction. Gwenaëlle Briand & Cloonan [19]; Kampfer, Leischnig, et al. [20], and Peck and Childers [21] showed how haptic information can influence consumer product evaluations. The findings by Ranaweera et al. [22] and Krishna et al. [13] highlight that tactile impressions, specifically the interaction between surface texture and weight, significantly shape consumer evaluations and willingness to purchase, particularly when aligned with individual differences in the need for touch, underscoring the critical role of haptic cues in driving product impressions and brand perception. The inability to physically interact with products is a major factor contributing to high return rates in online shopping [23–24]. Studies indicate that consumers who can touch products are more likely to make a purchase [25–26], and haptic priming further shapes their preferences [17,27,28]. In response to the lack of tactile interaction in e-commerce, marketers have implemented strategies such as sensory descriptions [29]. Despite these efforts, further research is needed to understand how tactile impressions influence consumers’ WTP for specific products.

A recent study provides strong evidence that links tactile impressions to consumer preferences; however, their roles in pricing strategies remain underexplored [14]. Kadoya et al. [14] found that consumers are generally willing to pay a premium for enhanced smartphone cover textures, with variations based on factors such as age and risk preferences. However, the limited sample size of 139 university students restricts its generalizability. Additionally, it did not account for behavioral economic factors, such as time discounting, which can influence consumers’ WTP. To address these limitations, this study builds on previous research by incorporating a larger and more diverse sample, which allows for a broader analysis across different socioeconomic backgrounds, age groups, and purchasing habits, enhancing the external validity of our findings. Moreover, we introduce time and hyperbolic discounting as additional explanatory variables, drawing on behavioral economics theories to better understand how consumers evaluate immediate versus future costs and benefits when making purchasing decisions.

Time discounting refers to individuals’ tendency to value immediate rewards more than delayed ones, even when the delayed rewards are objectively larger [30]. This concept is crucial in pricing decisions, as consumers may perceive the future benefits of tactile impressions differently depending on their time preferences. While traditional economic models assume exponential discounting, where future values are consistently discounted over time, studies have shown that individuals often exhibit hyperbolic discounting. This leads to inconsistencies in decision-making, as the discount rate decreases over time [31–32]. The influence of hyperbolic discounting on product purchase decisions have been evident since long [33–36]. Several studies Werthschulte and Löschel [37], Xu et al. [38], Calcott, P., & Petkov [39], Nguyen [40], Angeletos et al. [41] and Meier and Sprenger [42] showed that present-biased consumers tend to overspend on current consumption and reduce consumption spending around retirement [37–42]. Richards and Green [43] found that hyperbolic discounting affect the valuation of environmentally sustainable goods which produce benefits in the long-term. Overall, hyperbolic discounting is an important psychological trait that can be leveraged to design marketing strategies focused on user experience such as offering immediate rewards, limited-time offers, and breaking down long-term goals into smaller, more manageable steps [44–45]. In this context, hyperbolic discounting may significantly shape how tactile impressions affect price decision. When consumers physically interact with a smartphone cover, consumers often experience immediate sensory gratification such as texture, grip, and material feel, which elicits an emotional response and enhances perceived value. This aligns with Peck and Shu’s [26] findings that tactile interaction improves psychological ownership, leading to a greater WTP. However, hyperbolic discounting may prioritize immediate gratification over long-term consideration, leading to impulsive purchases at higher prices. Conversely, if decision-making is delayed, consumers may downplay tactile attributes and instead emphasize functional consideration, such as durability or price.

Beyond behavioral traits, demographic and socioeconomic factors also shape the valuation of tactile impressions. Experimental studies have documented sex-based differences in tactile sensitivity and material preferences, suggesting that men and women respond differently to texture. For example, women tend to have higher tactile acuity, due to biological factors such as smaller finger size and a greater density of mechanoreceptors, enabling more precise texture discrimination [45–49]. Moreover, consumer research shows that women are often more emotionally responsive to sensory cues, while men typically prioritize functionality unless promoted to evaluate hedonic evaluation [50]. These perceptual and psychological differences may lead to divergent patterns in willingness to pay for texture-enhanced products. Marital status may also affect valuation through variation in lifestyle, household budgeting dynamics [51]. Furthermore, income and wealth levels shape sensory valuation through resource allocation tendencies and time orientation. According to Boon-Falleur et al. [52], individuals with greater financial resources tend to adopt a more future-oriented mindset, show greater tolerance for variance, and invest more readily in low-priority or hedonic features, such as premium textures. Together, demographic and socioeconomic traits have the potential to influence consumers’ willingness to pay for surface textures.

This study examines how tactile impressions influence consumers’ WTP for smartphone covers while integrating behavioral economic factors that may affect decision-making. Specifically, we assess the extent to which consumers value tactile impressions of smartphone covers and whether this valuation varies across demographic and socioeconomic groups. Additionally, we investigate the role of time and hyperbolic discounting in the WTP for tactile impressions, examining whether individuals with a stronger present bias are less willing to pay a premium for enhanced textures. Additionally, we analyze the relationship between time consistency in decision-making and WTP, determining whether individuals with stable preferences over time are more likely to invest in higher-quality tactile experiences. We also compare the findings with those of previous research [14] to evaluate consistency and offer new insights into how product texture interacts with consumer psychology and economic preferences.

The novelty of this study lies in its interdisciplinary approach that integrates sensory marketing with behavioral economics to examine how tactile impressions influence consumer WTP. While previous research treated tactile impressions as an isolated factor in consumer choice [7,9,10,17,18], this study positions tactile perception within a broader decision-making framework. By incorporating time and hyperbolic discounting models, we explore whether consumers prefer immediate or long-lasting tactile benefits. This approach elucidates consumer preferences, contributing to marketing theory and the development of pricing strategies. Moreover, by extending theoretical justifications to demographic and socioeconomic factors, we also provide a more comprehensive understanding of who values texture-based differentiation and why. This richer theoretical foundation enables a more nuanced interpretation of our results and better positions our study within the existing body of literature on sensory perception, economic decision-making, and market segmentation.

This study makes several contributions to existing literature. First, it extends prior research on the WTP for tactile impressions with a larger and more diverse sample, addressing the generalizability concerns identified by Kadoya et al. [14]. Second, it introduces time and hyperbolic discounting as explanatory variables, offering new insights into how intertemporal preferences shape consumer valuation of product features. Third, it provides empirical evidence of the intersection of sensory perception and economic decision-making, demonstrating how tactile impressions can be strategically leveraged in pricing models to enhance consumer engagement and product appeal.

The remainder of this paper is organized as follows. The Materials and Methods section describes the data sources, procedures, and analytical approaches used in this research. The Results section reports the main findings, followed by the Discussion, which interprets these results in the context of existing literature and study objectives. The Limitations section acknowledges the study’s constraints, and finally, the Conclusion summarizes the key insights and implications.

## Materials and methods

### Sampling and participants

The experiment to assess consumers’ WTP for smartphone covers with different tactile impressions was conducted at Youme Town, the largest and most frequented shopping mall in Higashi-Hiroshima, Japan, between January 23 and February 8, 2025. Youme Town is one of the region’s largest and busiest commercial complexes, offering groceries, household goods, fashion, electronics, restaurants, and more. This wide product ix attracts a demographically diverse customer base- from students and working adults to families and retirees. Consequently, the site provided an ideal natural environment for observing authentic consumer behavior while maintaining controlled sensory conditions. Participants were recruited through open invitations at the store without demographic restrictions, ensuring unbiased participation. Because the customer reflects the socioeconomic and lifestyle diversity typical of mid-sized Japanese urban areas, the setting enhanced both ecological validity and the generalizability of the findings. The study followed a structured two-stage sampling process to ensure a diverse and representative group of participants.

In the first stage, an open invitation was extended to shoppers entering the shopping mall, inviting them to participate in a consumer study. To minimize response bias, the study’s purpose was not disclosed at this stage. Interested individuals voluntarily expressed their willingness to participate. In the second stage, all prospective participants were fully informed about the study’s objectives, methodology, and procedures. After receiving this information, participants provided their informed consent before taking part in the experiment. This recruitment process ensured that participation was entirely voluntary, free from coercion, and not influenced by the data collection team.

The final sample consisted of 387 respondents, meeting the minimum sample size required to achieve a 95% confidence level with a 5% margin of error. This ensures an unbiased estimation of consumers’ WTP for tactile impressions, enabling reliable statistical analysis of the factors that influence valuation across different demographic and behavioral groups.

The study was approved by the Ethics Committee of Hiroshima University, Japan (Approval Number: HR-LPES-002352). A copy of the original ethics approval letter is included as a supporting file. Prior to participation, all respondents were informed about the objectives of the study, the voluntary nature of their involvement, and their right to withdraw at any time. We obtained written informed consent from all participants. No minors were involved in this study, and no part of the consent requirement was waived by the ethics committee.

Data were collected using a structured questionnaire, which gathered information on tactile perceptions of smartphone covers and demographic and socio-economic characteristics such as sex, age, marital status, living arrangements, household income, assets, and preferences related to risk and time. The primary objective was to measure consumers’ WTP for smartphone covers with different tactile impressions, ensuring that participants did not consider shape, function, or color in their evaluations.

To accurately measure WTP, the study employed the contingent valuation method (CVM) with an open-ended question format, asking respondents directly how much they would be willing to pay for each type of smartphone cover [53–54]. As establishing a reference price is essential when using open-ended WTP measures [53,55,56], the study relied on external market prices as benchmarks [56–59]. A smartphone cover from a 100-yen shop (equivalent to a dollar store in North America) was selected as the reference product because of its widespread availability and affordability. As of 2024, over 10,000 such stores existed across the country, making these covers accessible and commonly used by most consumers [60–62]. Furthermore, smartphone covers from 100-yen shops are widely regarded as viable substitutes for more expensive alternatives on the market, helping to minimize estimation bias when measuring WTP. For this study, a smartphone cover from DAISO was used, as the chain is widely recognized as a flagship representative of 100-yen shops in Japan. Known for its extensive product range, consistent quality, and nationwide presence, DAISO exemplifies the value and accessibility of the 100-yen shop model.

**Fig 1:**
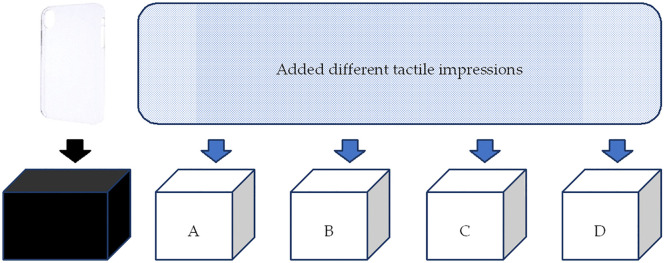
Smartphone covers with and without surface textures in the boxes.

**Fig 2:**
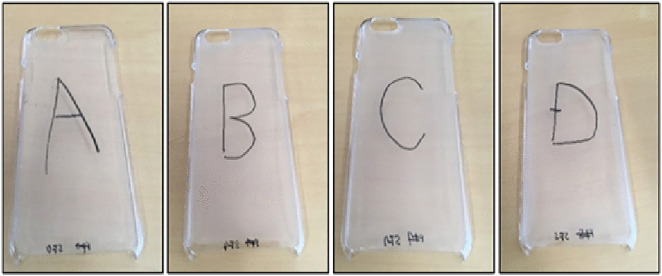
Smartphone covers with different surface roughness.

To establish a basis for comparison, this study used both a reference product and a reference price. Participants were provided with a standard plastic smartphone cover from a 100-yen shop as the reference product, along with four additional smartphone covers labeled A, B, C, and D, each featuring a distinct surface texture (Fig 1 and Fig 2). These textures were designed using different materials or variations of the same material in terms of smoothness, height, slipperiness, dampness, granularity, stickiness, and dryness. These attributes were based on prior research that identified the key surface roughness parameters affecting tactile impressions. Table 1 presents the surface-roughness parameters used in the experiments.

**Table 1:** Surface roughness parameters used in the experiment.

Surface Type	Arithmetic Mean Roughness (Ra)	Maximum Height (Rz)	Average Roughness (Rsm)
A	0.07	0.82	129
B	1.69	9.53	99
C	0.65	4.45	162
D	1.06	10.63	179

The selection of surface textures A–D was informed by both theoretical and practical considerations [63,64]. We aimed to capture a range of tactile sensations that are representative of materials used in commercial smartphone accessories. The surface roughness parameters (Ra, Rz, Rsm) were derived from prior research in tactile perception and haptics, which identified these as key indicators of perceived texture intensity and quality [14,63,64]. The four textures capture a meaningful range of surface-roughness characteristics, based specifically on the values of arithmetic mean roughness (Ra), maximum height (Rz), and mean spacing of profile elements (Rsm). Surface A (Ra = 0.07 µm, Rz = 0.82 µm, Rsm = 129 µm) exhibits extremely low roughness, while Surface B (Ra = 1.69 µm, Rz = 9.53 µm, Rsm = 99 µm) shows substantially higher peak variation and denser profile features. Surfaces C and D occupy intermediate positions, with differing combinations of moderate Ra and Rz values and relatively wider Rsm spacing. This range enables us to explore perceptual and evaluative differences across tactile stimuli that vary not only in roughness magnitude but also in surface feature density. By grounding the selection in metrological data and known application contexts, the textures provide an appropriate foundation for investigating consumer response to surface variation. Additionally, pilot testing was conducted to ensure that the selected textures were perceptibly distinct to the average consumer. Participants in the pilot indicated that the covers resembled real-world tactile surfaces commonly found in smartphone. This ensured ecological validity while maintaining experimental control.

To assess tactile impressions, the reference smartphone and textured covers were placed in designated boxes. Participants were instructed to physically feel each cover and then indicate how much they would be willing to pay for each, relative to the reference cover, which did not feature any texture. The WTP was measured under two price conditions: first, when the reference cover was priced at 100 yen, representing a low-cost smartphone cover, and second, when the reference cover was priced at 1,000 yen, representing an average market-priced smartphone cover. Participants were asked to compare each textured cover to the reference product and indicate their WTP under both price conditions. To ensure the validity of the responses, participants were explicitly instructed not to consider the shape, function, or color of the smartphone covers. This was to ensure that any variation in WTP could be attributed solely to the surface texture. As all smartphone covers used in the study were identical in shape, function, and color, any difference in WTP could be attributed only to surface texture.

**Fig 3:**
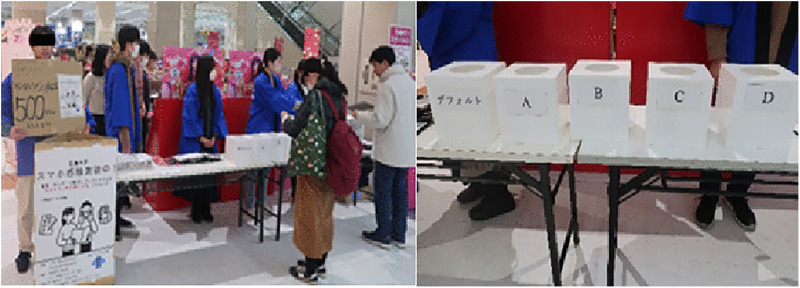
Participants evaluated the smartphone covers.

The experimental setup shows how participants evaluated the smartphone covers with and without surface textures (Fig 3). The inclusion of two reference prices allowed for an investigation into how participants’ valuation of tactile impressions varied with higher price expectations. This approach also accommodated different levels of price sensitivity among participants, with the 100-yen reference price targeting budget-conscious consumers and the 1,000-yen reference price targeting those considering mid-range to premium products.

### Measurement

To examine the factors influencing consumers’ WTP for smartphone covers with different tactile impressions, this study follows Kadoya et al. [14] for consistency with the existing literature on consumer valuation and economic behavior. The dependent variables represent the price that the respondents were willing to pay for smartphone covers with different surface textures under the two reference price conditions. Specifically, the WTP was recorded separately for the four surface textures (A, B, C, and D) when the reference price was 100 yen (ap100, bp100, cp100, and dp100) and 1,000 yen (ap1000, bp1000, cp1000, and dp1000). Respondents were asked to evaluate the surface texture without considering other attributes of the product, such as shape, function, or color.

The independent variables included demographic and socioeconomic factors that could influence WTP. These variables capture individual characteristics, such as sex, age, marital status, living arrangements, education level, and student status. Socioeconomic conditions were accounted for using household income and household assets, whereas behavioral factors were represented by smartphone usage, risk preference, impatience, and hyperbolic discounting. The inclusion of these variables allows for a comprehensive analysis of how consumer characteristics impact WTP for tactile impressions. By incorporating a diverse set of factors, this study provides insights into how demographic, economic, and behavioral traits shape consumer preferences for smartphone cover texture. Table 2 shows definitions and measurement of varaibles used in this study.

**Table 2:** Variable definitions.

Variables	Definition
Dependent Variable	
ap100	The amount respondents were willing to pay for a smartphone cover with an A-type surface texture, disregarding its shape, function, or color, when the reference price was set at 100 yen (unit: yen)
ap1000	The amount respondents were willing to pay for a smartphone cover with an A-type surface texture, disregarding its shape, function, or color, when the reference price was set at 1000 yen (unit: yen)
bp100	The amount respondents were willing to pay for a smartphone cover with a B-type surface texture, disregarding its shape, function, or color, when the reference price was set at 100 yen (unit: yen)
bp1000	The amount respondents were willing to pay for a smartphone cover with a B-type surface texture, disregarding its shape, function, or color, when the reference price was set at 1000 yen (unit: yen)
cp100	The amount respondents were willing to pay for a smartphone cover with a C-type surface texture, disregarding its shape, function, or color, when the reference price was set at 100 yen (unit: yen)
cp1000	The amount respondents were willing to pay for a smartphone cover with a C-type surface texture, disregarding its shape, function, or color, when the reference price was set at 1000 yen (unit: yen)
dp100	The amount respondents were willing to pay for a smartphone cover with a D-type surface texture, disregarding its shape, function, or color, when the reference price was set at 100 yen (unit: yen)
dp1000	The amount respondents were willing to pay for a smartphone cover with a D-type surface texture, disregarding its shape, function, or color, when the reference price was set at 1000 yen (unit: yen)
Independent Variable	
Sex	A binary variable where male is coded as 1 and female as 0
Age	A continuous variable representing the age of respondents
Age squared	A continuous variable representing the square of age of respondents
Spouse	A binary variable where spouse is coded as 1 and 0 otherwise
Live alone	A binary variable where living alone is coded as 1 and 0 otherwise
University degree	A binary variable where having a university degree is coded as 1 and 0 otherwise
Student	A binary variable where an undergraduate or graduate student is coded as 1 and 0 otherwise
Household income	A continuous variable representing the annual household income of respondents
Household asset	A continuous variable representing the balance of household assets of respondents
Usage	A continuous variable representing the average time of usage of smartphone per day of respondents
Risk preference	Continuous variable: risk of rain preference (usually, when you go out, how high does the probability of rain have to be before you take an umbrella?)
Impatience	A continuous variable representing respondents’ average standardized values of the elicited discount rates (DR1 and DR2)
Hyperbolic discounting	A binary variable where hyperbolic discounter is coded as 1 and 0 otherwise

### Descriptive statistics

Table 3 presents the descriptive statistics for the main variables used in this study based on a sample size of 387 respondents. This table reports the mean, standard deviation, and minimum and maximum values for each variable, providing insights into the distribution of responses. The dependent variables represent WTP for smartphone covers with four different surface textures (A, B, C, and D) under two pricing conditions: 100 yen (low reference price) and 1,000 yen (high reference price). The mean WTP values range from 352.53 yen (bp100) to 1582.86 yen (dp1000), indicating that respondents, on average, were willing to pay higher amounts for some textures. However, the standard deviations were considerably large for all the WTP variables, suggesting high variability in responses, with some individuals willing to pay significantly more than others. The minimum WTP for all textures and pricing conditions is 0, indicating that some respondents were unwilling to pay for certain types of covers. However, the maximum values range from 60,000 yen (bp100) to 168,000 yen (dp1000), indicating that some participants assigned extremely high values to particular textures.

**Table 3:** Descriptive statistics.

Variable	Mean	Std. Dev.	Min	Max
Dependent Variable				
ap100	388.26	4073.62	0	80000
ap1000	1220.80	4528.35	0	88000
bp100	352.53	3061.07	0	60000
bp1000	1187.02	3429.69	0	66000
cp100	473.28	5086.79	0	100000
cp1000	1338.48	5166.46	0	101000
dp100	640.30	8134.68	0	160000
dp1000	1582.86	8618.48	0	168000
Sex	0.41	0.49	0	1
Age	38.30	17.22	18	87
Age squared	1762.74	1527.96	324	7569
Spouse	0.27	0.45	0	1
Live alone	0.38	0.49	0	1
University degree	0.43	0.50	0	1
Student	0.46	0.50	0	1
Household income	5886305	4217837	1000000	20000000
Household asset	13100000	18600000	2500000	100000000
Usage	285.23	176.69	0	1230
Risk preference	0.57	0.24	0	1
DR1	1.79	1.35	−0.1	3.0
DR2	1.83	1.35	−0.1	3.0
Impatience	0.00	0.93	−1.41	0.88
Hyperbolic discounting	0.10	0.30	0	1
	387			

The independent variables capture the demographic, socioeconomic, and behavioral characteristics of the respondents. Sex is a binary variable, where male is coded as 1 and female as 0, with a mean of 0.41, indicating that 41% of the participants were male. The average age of the respondents is 38.3 years, with a standard deviation of 17.22, ranging from 18 to 87 years, reflecting a relatively diverse age distribution. Age-squared is included to capture the potential nonlinear effects of age on WTP, with a mean of 1762.74. The variable indicating whether a respondent had a spouse has a mean of 0.27, suggesting that 27% of the respondents were married. The variable for living alone has a mean of 0.38, meaning that 38% of the respondents lived alone. The proportion of respondents with university degrees is 43%, and students comprised 46% of the sample. Socioeconomic variables exhibit substantial variation. Household income has a mean of 5,886,305 yen and a standard deviation of 4,217,837 yen, ranging from 1,000,000 yen to 20,000,000 yen, indicating considerable income differences among respondents. Household assets show even greater dispersion, with a mean of 13,100,000 yen but ranging from 2,500,000 yen to 100,000,000 yen.

Regarding smartphone usage and behavioral characteristics, the average smartphone usage time is 285.23 min per day (approximately 4.75 h), but this varies widely from 0 to 1,230 min. Risk preference, measured on a scale between 0 and 1, has a mean of 0.57, suggesting a moderate level of risk-taking among participants. DR1 and DR2, which measure decision-related behavior, have similar means of 1.79 and 1.83, respectively. Impatience has a mean of 0.00 and a standard deviation of 0.93, indicating variation in how respondents value immediate rewards. Hyperbolic discounting, which assesses whether respondents discount future rewards more steeply, has a mean of 0.10, with a minimum of 0 and a maximum of 1. Thus, while some respondents exhibit this behavior, others do not.

### Methods

This study employed ordinary least squares (OLS) regression to analyze the factors influencing WTP for smartphone covers with different tactile impressions. OLS was selected because it provides a straightforward interpretation of the relationship between explanatory and dependent variables, making it suitable for analyzing how demographic, socioeconomic, and behavioral factors affect WTP. As WTP is a continuous measure obtained from an open-ended question format, this method allows for direct estimation while ensuring interpretability. To address potential skewness in the WTP distribution and improve the normality of the residuals, the dependent variable was log-transformed before estimation.

Regression analysis was conducted separately for each price condition of the four reference smartphone covers A, B, C, and D, and 100 yen and 1,000 yen to examine whether consumers’ valuations of tactile impressions varied across different pricing scenarios. The regression equation used in this study is as follows:

Log (WTP) = β0 + β1 (Sex) + β2 (Age) + β3 (Age2) + β4 (University Degree) + β5 (Student) + β6 (Spouse) + β7 (Live Alone) + β8 log(Household Income) + β9 log(Household Assets) + β10 (Usage) + β11 (Risk Preference) + β12 (Impatience) + β13 (Hyperbolic Discounting) + ∊i

where log (WTP), the dependent variable, represents the natural logarithm of WTP for smartphone covers with different tactile impressions. The independent variables included sex, age, age squared, university degree, student, spouse, living alone, log of household income, log of household assets, usage, risk preference, impatience, and hyperbolic discounting. Finally, the error term represents unobserved factors.

We assessed the presence of multicollinearity among the independent variables by conducting a variance inflation factor (VIF) test. Multicollinearity arises when independent variables are highly correlated, potentially distorting the estimated coefficients and making it difficult to determine the individual effects of each predictor on WTP. The VIF test results indicated that all values were well below the commonly accepted threshold of 7, suggesting that multicollinearity is not a concern in our model. Although the VIF test results are not presented here, they are available upon request. This confirms that the included variables independently explain the variations in WTP without significant redundancy in information.

## Results

### Descriptive statistics for the WTP for various surface textures

Descriptive statistics for WTP for different surface textures and reference prices are presented in Table 3. The results showed substantial variation in the amount that respondents were willing to pay, reflecting the differences in consumer preferences for tactile impressions.

For the A-type surface texture, the respondents were willing to pay an average of 388.26 yen when the reference price was 100 yen, with a standard deviation of 4,073.62 yen. The minimum WTP for this texture was 0 yen, and the maximum recorded value was 80,000 yen. When the reference price increased to 1,000 yen, the mean WTP increased to 1,220.80 yen, with a higher standard deviation of 4,528.35 yen and a maximum WTP of 88,000 yen. The increase in both the mean and maximum values at a higher reference price suggests that the respondents were willing to pay a premium for the A-Type A texture when the pricing anchor was higher.

For the B-type surface texture, the mean WTP at the 100-yen reference price was 352.53 yen, with a standard deviation of 3,061.07 yen. The minimum WTP was 0 yen and the maximum reached 60,000 yen. When the reference price increased to 1,000 yen, the mean WTP increased to 1,187.02 yen, with a standard deviation of 3,429.69 yen and a maximum value of 66,000 yen. Similar to the A-type, the increase in mean WTP at a higher reference price indicates that consumers adjusted their valuation upward in response to the price framework.

Regarding the C-type surface texture, respondents reported a mean WTP of 473.28 yen when the reference price was 100 yen, with a standard deviation of 5,086.79 yen. The minimum recorded value was 0 yen, while the maximum was 100,000 yen; the highest maximum WTP was recorded among all textures at the lowest price reference. At the 1,000-yen reference price, the mean WTP increased to 1,338.48 yen, with a standard deviation of 5,166.46 yen, and a slightly higher maximum value of 101,000 yen. Thus, some consumers placed an extremely high value on this particular tactile impression.

Concerning the D-type surface texture, the 100-yen reference price yielded the highest mean WTP among all the textures, with an average of 640.30 yen and a standard deviation of 8,134.68 yen. The minimum WTP was 0 yen, whereas the maximum recorded value was 160,000 yen, indicating that some respondents placed exceptionally high values on this texture. At the 1,000-yen reference price, the mean WTP further increased to 1,582.86 yen, with a standard deviation of 8,618.48 yen and a maximum value of 168,000 yen, the highest WTP reported for all textures and price levels. Therefore, the D-type texture was the most valued among the respondents, particularly when the reference price was higher.

Across all textures, the results indicate that the mean WTP increased consistently when the reference price was 1,000 yen instead of 100 yen. The variability, reflected in standard deviations and maximum values, also increased with higher reference prices. Thus, respondents adjusted their WTP based on the reference price provided. The substantial differences between the minimum and maximum WTP values indicate a diverse range of consumer preferences, with some respondents being highly price sensitive, while others exhibited a much greater WTP for specific tactile impressions.

### Regression results

The regression results for the eight models that estimate the WTP for smartphone covers with different surface textures under two reference price conditions (100 yen and 1,000 yen) are presented in Table 4. Each model corresponds to a specific surface texture (A, B, C, or D) and reference price level, with the dependent variable being the log-transformed WTP. The findings indicate the varying influences of demographic, socioeconomic, and behavioral factors on consumer WTP across different textures and price conditions.

**Table 4:** Regression results.

Variables	Log ap100	Log ap1000	Log bp100	Log bp1000	Log cp100	Log cp1000	Log dp100	Log dp1000
Sex	−0.0012	0.0759	0.0144	0.2168**	0.0888	0.2118***	0.0739	0.2318**
	(0.106)	(0.091)	(0.110)	(0.086)	(0.101)	(0.079)	(0.122)	(0.097)
Age	0.0138	0.0057	0.0244	0.0486	0.0266	0.0223	−0.0120	0.0380
	(0.022)	(0.029)	(0.023)	(0.035)	(0.022)	(0.029)	(0.026)	(0.028)
Age squared	−0.0001	−0.0001	−0.0002	−0.0005	−0.0003	−0.0003	0.0001	−0.0005
	(0.000)	(0.000)	(0.000)	(0.000)	(0.000)	(0.000)	(0.000)	(0.000)
University degree	−0.1547	0.0600	−0.0396	0.1237	−0.0247	0.0833	−0.0093	0.0388
	(0.146)	(0.123)	(0.133)	(0.124)	(0.141)	(0.115)	(0.158)	(0.124)
Student	−0.0163	0.1262	−0.0314	0.2517	0.0222	0.1445	−0.240	−0.0511
	(0.176)	(0.151)	(0.182)	(0.209)	(0.180)	(0.143)	(0.237)	(0.172)
Spouse	−0.2254	−0.0481	−0.4766**	−0.2255	−0.3622*	−0.1817	−0.4861**	−0.4457**
	(0.218)	(0.189)	(0.185)	(0.166)	(0.200)	(0.164)	(0.223)	(0.191)
Live alone	−0.2322	−0.0822	−0.2165	0.1520	−0.126	0.0088	−0.2789	−0.0614
	(0.213)	(0.173)	(0.208)	(0.203)	(0.221)	(0.173)	(0.246)	(0.187)
Log of Household income	−0.0575	0.0524	−0.1217	−0.0048	−0.1245*	0.0138	−0.0817	−0.0169
	(0.069)	(0.063)	(0.078)	(0.063)	(0.073)	(0.060)	(0.079)	(0.070)
Log of Household asset	0.0178	−0.0145	0.0914	−0.0317	0.0742	0.0350	0.0589	0.0804
	(0.061)	(0.055)	(0.064)	(0.078)	(0.062)	(0.051)	(0.076)	(0.058)
Usage	0.0003	−0.0002	0.0001	−0.0002	0.0003	0.0002	0.0003	0.0001
	(0.000)	(0.000)	(0.000)	(0.000)	(0.000)	(0.000)	(0.000)	(0.000)
Risk preference	−0.3300	−0.2407	−0.1992	−0.2253	−0.1163	−0.3342	−0.0230	−0.2240
	(0.265)	(0.251)	(0.268)	(0.229)	(0.253)	(0.223)	(0.321)	(0.279)
Impatience	−0.0820	−0.0352	−0.0821	−0.0675	−0.0459	−0.0482	−0.0257	−0.0486
	(0.062)	(0.052)	(0.062)	(0.047)	(0.057)	(0.044)	(0.069)	(0.060)
Hyperbolic discounting	0.1364	0.1127	0.2700*	0.3242***	0.2524	0.2040*	0.1774	0.2933**
	(0.115)	(0.151)	(0.146)	(0.112)	(0.156)	(0.112)	(0.145)	(0.130)
Constant	5.3248***	6.2657***	5.0328***	6.2908***	5.1668***	5.7415***	5.6119***	5.3085***
	(1.084)	(1.231)	(1.194)	(1.135)	(1.229)	(1.096)	(1.341)	(1.212)
Observations	387	387	387	387	387	387	387	387
R-squared	0.0296	0.0315	0.0414	0.0920	0.0352	0.1062	0.0288	0.0883
Adjusted R-squared	−0.0042	−0.0023	0.0080	0.0604	0.0016	0.0751	−0.0050	0.0565
Log likelihood	−561.3	−511.5	−563.8	−509.7	−555.1	−480.8	−615.3	−553.1
F-value	0.892	0.626	1.312	2.643	1.015	2.607	1.401	2.481

Note: Robust standard errors are shown in parentheses. *** p < 0.01, ** p < 0.05, * p < 0.10.

Sex exhibits a significant positive effect on WTP for B-, C-, and D-type textures at the 1,000-yen reference price level, suggesting that males are willing to pay more for these textures than females. The coefficient for sex is statistically significant at the 5% level for B-type (0.2168) and D-type (0.2318) texture and at the 1% level for C-type (0.2118) texture when the reference price is 1,000 yen. However, sex does not significantly affect WTP for the A-type texture or any of the textures at the 100-yen price level.

Spouse status shows a significant negative effect on WTP, particularly for B- and D-type texture at both reference price levels and for the C-type at the 100-yen level. The coefficients for B-type at 100 yen (−0.4766) and D-type at 100 yen (−0.4861) are significant at the 5% level, whereas the coefficient for C-type at 100 yen (−0.3622) is significant at the 10% level. Thus, respondents with spouses are generally less willing to pay for smartphone covers of certain textures.

Log of household income significantly negatively affects WTP for the C-type texture at the 100-yen price level, with a coefficient of −0.1245 (p < 0.10), indicating that individuals with higher household income are less willing to pay for this texture at a lower reference price. However, no significant income effects are observed for the other textures.

Hyperbolic discounting has a notable impact on WTP, particularly for B- and C-type texture at the 1,000-yen level. The coefficient for B-type at 1,000 yen (0.3242) is significant at the 1% level, whereas for C-type at 1,000 yen (0.2040), the effect is significant at the 10% level. Additionally, for D-type at 1,000 yen, hyperbolic discounting is significant at the 5% level (0.2933), suggesting that individuals who exhibit hyperbolic discounting are more likely to assign higher WTP values to the premium price level.

Other demographic variables, including age, student status, and living alone, did not exhibit significant effects across the models. Additionally, the log of household assets, smartphone usage, risk preference, and impatience showed no statistically significant influence on the WTP for any of the texture or price conditions.

To assess the robustness of our findings, we re-estimated the regression models after excluding the top 1% of extreme WTP values. The results remained largely consistent with the original estimates, indicating that our findings are not driven by outliers. As shown in Appendix 1, key predictors such as sex, spousal status, income, and hyperbolic discounting remained statistically significant in explaining willingness to pay for different surface textures. These robustness checks reinforce the validity of our main conclusions.

Overall, the findings suggest that sex, marital status, income, and hyperbolic discounting influence the WTP for smartphone covers with different tactile impressions, with these effects being more pronounced at higher reference price levels.

## Discussion

This study provides key insights into how tactile impressions affect consumers’ WTP for smartphone covers across various surface textures and reference prices. The findings support prior research in sensory marketing and behavioral economics, reinforcing the role of texture in shaping product valuation [9,17,18]. Descriptive statistics indicate notable variation in WTP across textures. D-type textures received the highest mean WTP (640.30 yen at the 100-yen reference price; 1,582.86 yen at the 1,000-yen reference price), whereas B-type textures received the lowest (352.53 yen and 1,187.02 yen, respectively). These results suggest that some textures are perceived as more desirable, potentially due to associations with comfort, grip, or superior quality. The consistent increase in WTP at the 1,000 yen reference price across all textures reflects consumers’ susceptibility to pricing cues, consistent with anchoring theory [65,66].

Regression analysis reveals that sex significantly influences WTP for B-, C-, and D-type textures at the 1,000-yen reference price, with males generally exhibiting a higher WTP. This may be attributed to sex differences in anchoring effects and perceived value. Prior research suggests that women are more susceptible to anchoring biases, relying more heavily on initial price cues [49], which may lead them to focus on base prices rather than product features such as texture. Conversely, men may be more attuned to the added value of premium textures and thus more willing to pay for them—especially at higher reference prices, where perceived value increases [48]. No significant sex effect was found for A-type textures, indicating a more neutral appeal across sexes.

The negative effect of spouse status on WTP, particularly for B- and D-type textures at both price levels, suggests that married individuals may be more price-sensitive or less influenced by tactile impressions than single individuals. This may reflect household financial decision-making dynamics, wherein married consumers prioritize functional attributes over sensory appeal. Savitha and Sathyanarayan [51] found that although married individuals do purchase luxury goods, their decisions are more influenced by family considerations, leading them to favor products offering functional value and durability. Accordingly, married respondents may perceive surface texture, a primarily aesthetic feature, as an unnecessary expense and prefer practical benefits instead.

Household income (log-transformed) negatively affects WTP for C-type textures at the 100-yen reference price, indicating that higher-income consumers are less inclined to pay for sensory features in low-priced products. This may reflect a perception that budget smartphone covers are low-status goods, thus reducing the perceived value of their tactile attributes. However, this effect does not persist at the 1,000-yen price level, suggesting that higher-income consumers are more responsive to tactile impressions when the product aligns with premium quality expectations.

Hyperbolic discounting significantly increases WTP for B-, C-, and D-type textures at the 1,000-yen reference price. Consumers exhibiting present bias tend to prioritize immediate gratification and are therefore more willing to pay for textures that offer immediate tactile satisfaction. This supports Laibson’s [31] hyperbolic discounting model, which posits that individuals overvalue immediate rewards. It also aligns with the endowment effect [67], whereby physical interaction with a product increases its perceived value.

By contrast, smartphone usage and impatience do not significantly influence WTP, indicating that preferences for tactile impressions are driven more by intrinsic material preferences than by usage patterns or time preferences. Unlike Kadoya et al. [14], who reported a positive relationship between risk preference and WTP, we found no such association. This discrepancy may stem from differences in sample composition or the inclusion of additional behavioral variables, such as hyperbolic discounting, which may have absorbed the explanatory power of risk preference.

From a theoretical perspective, these results reinforce the idea that tactile impressions are a crucial yet underexplored determinant of consumer valuation. Consistent with Peck and Shu’s [26] findings, our study confirms that haptic engagement enhances perceived product value, and it extends this notion by incorporating behavioral economic theories, such as hyperbolic discounting and reference-dependent preferences. Specifically, our findings suggest that present-biased consumers may be especially responsive to immediate sensory gratification, leading to elevated WTP for textured products. Furthermore, the observed price anchoring effect at the 1,000-yen reference point underscores the influence of contextual pricing in shaping perceived value. The elevated WTP associated with anchor aligns with established behavioral pricing theories, which posit that consumers often interpret prices as signals of superior quality [65]. Theoretically, these insights maybe generalized beyond smartphone covers to other hedonic and sensory-driven product, such as apparel, cosmetics, and food packaging, domain in which tactile impression plays an important role in consumer evaluation. For example, olfactory and gustatory clues also evoke immediate affective responses and could interact with temporal preferences in ways comparable to tactile stimuli. Future research may explore whether present-biased consumers exhibit similarly heightened responses to other sensory inputs when evaluating product value.

From a practical standpoint, the findings offer actionable guidance for marketers and retailers seeking to leverage sensory design and behavioral insights to influence consumer decision-making. For instance, premium pricing may be particularly effective for textures perceived as luxurious or distinctive, especially among male consumers or individuals with a tendency toward hyperbolic discounting. In physical retail environment, tactile engagement can be encouraged through sample zones or guided demonstrations, or interactive display that foster psychological ownership and potentially trigger the endowment effect, thereby increasing WTP. In online contents, where direct touch is unavailable, markets can simulate tactile experiences using vivid, sensory-rich language, metaphor-based descriptions, and augmented reality (AR) or haptic technologies that visually or physically approximate texture. By aligning tactile design with psychological drivers of valuation, firms can more effectively enhance perceived product quality and increase purchase likelihood.

Overall, this study contributes to the growing body of research on sensory marketing and behavioral pricing by demonstrating how tactile impressions, economic decision-making models, and reference pricing jointly influence consumer valuation. The results confirm that texture is not merely a passive design element but an active driver of WTP, shaped by both psychological and economic factors. Future research should examine how these findings apply to different product categories and explore whether alternative pricing mechanisms, such as dynamic pricing or bundling, can enhance consumer engagement with tactile impressions.

### Limitations

Although this study provides valuable insights into consumers’ WTP for tactile impressions in smartphone covers, several limitations should be acknowledged. First, the data were collected from a single shopping mall in Japan, which may restrict the external validity and generalizability of the findings. Consumer preferences and price sensitivity may vary across regions, retail contexts, or cultural environments, and future research should validate these results across more diverse settings. Second, while the use of the contingent valuation method (CVM) with open-ended questions is suitable for this context, self-reported WTP values are subject to hypothetical bias and strategic overstatement, which may affect their accuracy. Although efforts were made to minimize such biases such as using neutral framing and emphasizing anonymity, this limitation should be kept in mind when interpreting the findings. Third, some respondents reported extremely high WTP values (e.g., up to 168,000 yen), which could reflect outliers or unrealistic valuations. While the main regressions use log-transformed WTP to reduce the influence of these extreme values, we also conducted sensitivity analyses excluding the top 1% of responses, with consistent results. Finally, although the regression models revealed statistically significant associations between consumer characteristics and willingness to pay (WTP), the relatively low adjusted R-squared values suggest that a substantial proportion of the variation in WTP remains unexplained. This is not uncommon in behavioral economics and field experiments, where individual decision-making can be influenced by a wide array of unobserved factors. Nevertheless, it is important to acknowledge that additional variables may enhance the explanatory power of the models. For instance, psychological constructs such as materialism, need for uniqueness, or product involvement may influence consumer responses to tactile impressions. Similarly, brand perception, prior experience with tactile smartphone accessories, and environmental or emotional states during the experiment (e.g., mood, store ambiance, or social presence) may have shaped individual valuations. Future studies should consider incorporating such factors to provide a more comprehensive understanding of consumer valuation of texture-enhanced products.

## Conclusions

This study examines the influence of tactile impressions on consumers’ WTP for smartphone covers, addressing a gap in the literature where surface texture has been underexplored as a determinant of product valuation. While prior research has emphasized visual and auditory cues in shaping consumer preferences, tactile impressions have received limited attention. By integrating behavioral economic factors, such as time and hyperbolic discounting, alongside conventional socioeconomic variables, this study enhances the understanding of how surface textures affect consumer behavior.

Analyzing a diverse sample of 387 respondents, the study explored four distinct textures under two pricing conditions (100 yen and 1,000-yen reference prices) to assess the interaction between pricing anchors and tactile preferences in consumer decision-making. The findings reveal significant variation in WTP across textures, with D-type textures receiving the highest valuation and B-type textures the lowest. Sex, spouse status, and hyperbolic discounting were significant predictors of WTP. Men generally exhibited a higher WTP for B-, C-, and D-type textures at the higher reference price. Spouse status was negatively associated with WTP, suggesting that married individuals may be more price-sensitive or less influenced by tactile appeal. Additionally, hyperbolic discounters showed a higher WTP at the 1,000-yen price level, highlighting the role of immediate sensory gratification in purchasing decisions.

These findings have important implications for product design, pricing strategies, and marketing. Businesses can leverage pricing anchors to enhance consumer perception of premium textures, positioning textured products within higher price brackets to increase perceived value. Men and hyperbolic discounters represent key target segments for premium textured products, as they demonstrate a greater WTP for enhanced tactile experiences. Moreover, given the impact of immediate sensory feedback on WTP, retailers should allow consumers to physically engage with products before purchase, through in-store trials or sample displays. In e-commerce, the integration of sensory cues or touch-based technologies may help bridge the tactile gap, enhancing the online shopping experience. In summary, this study highlights the commercial relevance of tactile impressions and the importance of integrating sensory marketing with behavioral economic insights. Future research could explore the role of brand perception, product durability, and long-term user satisfaction in shaping WTP for textured products to elucidate the role of touch in consumer decision-making.

## Supporting information

S1 Data Final dataset used in the study. (DTA)
