# Correction: How surface texture affects consumers’ willingness to pay: Evidence from smartphone covers

**DOI:** 10.1371/journal.pone.0340319

**Published:** 2026-01-05

**Authors:** Yoshihiko Kadoya, Mostafa Saidur Rahim Khan, Honoka Nabeshima, Yu Kuramoto, Yuichi Kurita

The following information is missing from the Funding and Acknowledgement statement: This work was supported by the Council for Science, Technology and Innovation, ‘Cross-ministerial Strategic Innovation Promotion Program (SIP), Development of foundational technologies and rules for expansion of the virtual economy (JPJ012495)’ (Funding Agency: NEDO).
